# Chronic Inflammatory Demyelinating Polyneuropathy in Patients With Crohn’s Disease on Infliximab Therapy

**DOI:** 10.7759/cureus.19041

**Published:** 2021-10-25

**Authors:** Fahad Almuntashri, Kenan Binyaseen, Amal Alkhotani

**Affiliations:** 1 Department of Medicine, Umm Al-Qura University, Faculty of Medicine, Mecca, SAU; 2 Neurology Department, King Abdullah Medical City, Mecca, SAU

**Keywords:** anti-tnf-α treatment, infliximab, neurological complications, crohn’s disease, chronic inflammatory demyelinating polyradiculoneuropathy

## Abstract

Crohn’s disease (CD) is a chronic inflammatory disorder of the gastrointestinal tract that is frequently accompanied by systemic complications including peripheral neuropathies. Anti-tumor necrosis factor-alpha agents such as infliximab are an established treatment for immune-mediated diseases. However, they have been associated with adverse effects, including local reactions, infections, congestive heart failure, malignancies, and, rarely, they can cause neurological adverse effects on the central nervous system, as well as peripheral nervous system demyelination. Here, we report the case of an 80-year-old man with CD on infliximab therapy who presented with progressive weakness and numbness. A neurological examination and a nerve conduction study suggested chronic inflammatory demyelinating polyneuropathy (CIDP). The patient was started on oral corticosteroids and experienced transient improvement of his symptoms at the end of this course. Thus, CIDP could be one of the extraintestinal presentations of CD.

## Introduction

Crohn’s disease (CD) is a multifactorial, chronic, and progressive inflammatory bowel disease characterized by severe inflammation of the gastrointestinal tract [1]. It is frequently accompanied by extraintestinal manifestations, including uveitis, arthritis, ankylosing spondylitis, primary sclerosing cholangitis, and erythema nodosum, which impact 20-40% of patients [1]. Chronic inflammatory demyelinating polyneuropathy (CIDP) is the most common chronic autoimmune neuropathy characterized by progressive, symmetric, proximal, and distal muscle weakness, paresthesia, sensory dysfunction, and impaired balance, which evolve gradually over at least two months [2]. CIDP can be one of the extraintestinal presentations of CD [3]. The mechanism of this complication has not been well explained, and it is unclear whether the complication is immune-mediated or secondary to medication use [3]. Although several studies have shown a possible role of anti-tumor necrosis factor-alpha (anti-TNF-α) in the pathogenesis and development of central nervous system (CNS) demyelinating diseases, this relationship is less clear for demyelinating diseases of the peripheral nervous system (PNS) [4]. Here, we report the case of an 80-year-old man with longstanding CD on infliximab therapy who presented with progressive weakness secondary to CIDP.

## Case presentation

An 80-year-old Asian male who had been diagnosed with CD for more than 20 years and bronchial asthma for more than 30 years was referred to our hospital with complaints of upper and lower limb weakness and numbness, which had started eight months prior to presentation.

His condition had started as gradual and progressive bilateral and symmetrical weakness in the feet, which, along with numbness, had continued to progress in ascending manner up his legs until it reached the upper thighs. Afterward, the upper limbs became involved (numbness and weakness) and progressed distally and proximally until the mid-forearms. The weakness was associated with fatigability, although there was no ocular, bulbar, or respiratory involvement. The patient had no loss of bowel or bladder control, fever history, or symptoms of upper respiratory tract infection. Regarding his CD, initially, he was on azathioprine which was later changed to infliximab after an exacerbation of CD. When he was diagnosed with CIDP, we resumed azathioprine which was effective in controlling his CIDP. He had been stable with no new exacerbation.

Upon presentation, examination revealed a conscious; alert; oriented to time, person, and place; and coherent man with intact memory and fluent speech. Cranial nerve examinations were normal, although the motor examination revealed the symmetrical flaccid weakness of the upper and lower limbs, with 0 power in the distal lower limb and 3/5 power in the proximal lower limb muscles. In the upper limbs, he had 2/5 distal power and 4/5 proximal power with absent reflexes. He had hyperesthesia in the leg up to the knee and from the upper limb up to the mid-forearm. However, his proprioception and vibration senses were intact.

His workup showed normochromic normocytic anemia with hemoglobin of 9 g/dL. The rest of the parameters were normal. Cerebrospinal fluid examination showed protein of 100 mg/dL with normal cells, white blood cell count of 2 cells/µL, and glucose of 70 mg/dL. A spinal MRI was normal with no nerve roots enlargement, and protein electrophoresis was also normal. Vitamins B1, B6, and B12 were within normal limits. Autoantibodies such as anti-contactin-1 (CNTN1) and anti-nodal neurofascin antibodies (NF) were not tested as they were not available. Additionally, anti-nuclear antibodies (ANA), anti-dsDNA, and anti-neutrophil cytoplasmic antibody (ANCA) were negative. Moreover, hepatitis B and C serology were negative.

**Figure 1:**
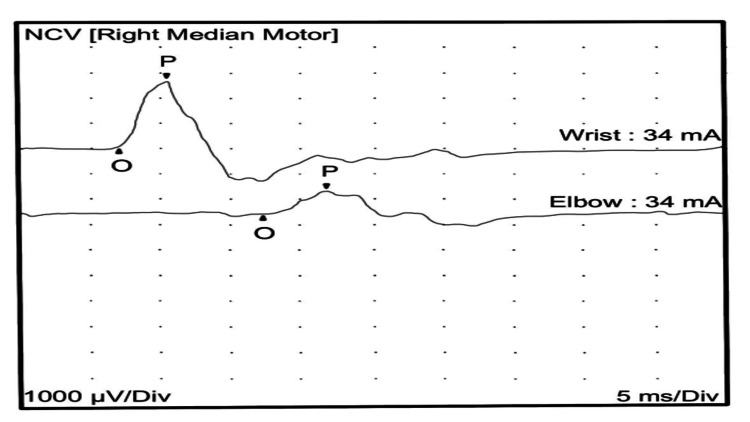
Right median nerve motor conduction showing reduced amplitude and conduction velocity with marked temporal dispersion. NCV: nerve conduction velocity

**Figure 2:**
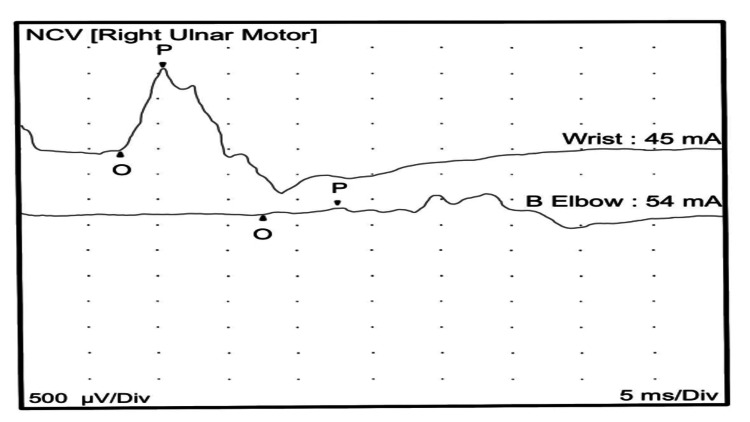
Right ulnar nerve motor conduction showing reduced amplitude and conduction velocity with marked temporal dispersion. NCV: nerve conduction velocity

A nerve conduction study of the upper and lower limb motor nerves showed prolonged distal onset latency, reduced amplitude, and decreased conduction velocity. The sensory nerve showed prolonged distal peak latency and decreased conduction velocity. The nerve conduction studies (Tables 1, 2), in keeping with severe demyelinating peripheral polyneuropathy, showed significantly reduced conduction velocity with marked temporal dispersion (Figures 1, 2).

**Table 1:** Summary of the anti-sensory test. The sensory nerve showed prolonged distal peak latency and decreased conduction velocity.

Site	NR	Peak (ms)	Normal peak (ms)	P-T amplitude (μV)	Normal P-T amplitude (μV)	Site 1	Site 2	Delta-P (ms)	Distance (cm)	Velocity (m/s)	Normal velocity (m/s)
Right ulnar anti-sensory (fifth digit)											
Wrist		9.3	<3.7	16.4	>15.0	Wrist	5th digit	9.3	14.0	15	>38

**Table 2:** Summary of the motor test. The motor study showed prolonged distal onset latency, reduced amplitude, and decreased conduction velocity.

Site	NR	Onset (ms)	Normal (ms)	O-P amplitude (mV)	Normal O-P amplitude (mV)	Site 1	Site 2	Delta-0 (ms)	Distance (cm)	Velocity (m/s)	Normal velocity (m/s)
Right median motor (abductor pollicis brevis)											
Wrist		7.2	<4.2	2.6	>5	Elbow	Wrist	10.2	24.0	24	>50
Elbow		17.4		0.9							
Right ulnar motor (abductor digiti minimi)											
Wrist		7.0	<4.2	1.6	>3	Below elbow	Wrist	10.2	24.0	24	>53
Elbow		17.2		0.2							

**Figure 3:**
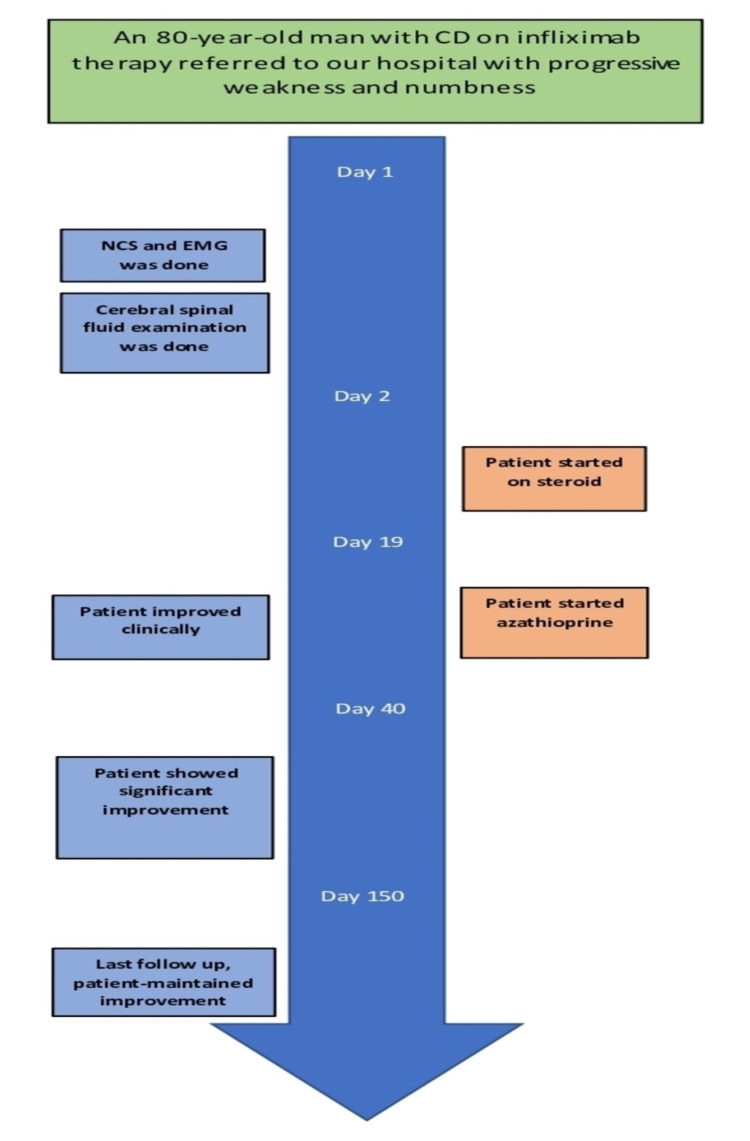
A timeline of the clinical events. NCS: nerve conduction study; EMG: electromyography

The patient refused treatment with intravenous immunoglobulin or plasma exchange even though they were better options concerning side effects. Instead, he was started on an oral steroid followed by the re-administration of azathioprine with significant improvement in his motor power as his follow-up motor examination revealed -4/5 in the distal lower limb and +4/5 in the proximal lower limb muscles. In the upper limbs, he had +4/5 distal power and 5/5 proximal power with normal reflexes. He became ambulatory with a cane and independent in his daily activities. His CD also remained stable. His treatment with infliximab continued as before after the options were discussed with both the patient and his treating gastroenterologist. Figure 3 summarizes the timeline of the clinical events.

## Discussion

CIDP is the most common immune-mediated chronic polyneuropathy. Although the incidence and prevalence of CIDP differ across studies, a meta-analysis showed a pooled incidence rate of 0.33 per 100,000 person-years and a pooled prevalence rate of 2.81 per 100,000 persons. Additionally, there is a male predominance, and the incidence and prevalence increase with advancing age [5]. Peripheral neuropathy is frequently reported as one of the complications of CD [1,6], but the exact prevalence is controversial [1,6]. Patients with CIDP classically present with symmetrical and progressive proximal and distal weakness [7]. Moreover, the presentation can be focal, multifocal, or sensorial predominantly. The diagnosis of CIDP is multifaceted, involving clinical history, physical examination, laboratory investigation, and electrodiagnostic studies, with the exclusion of other diseases with features similar to CIDP (e.g., hereditary demyelinating neuropathy, multifocal motor neuropathy, or *Borrelia burgdorferi* infection). Although several criteria have been proposed for CIDP diagnosis, the European Federation Neuropathy Society/Peripheral Neuropathy Society (EFNS/PNS) is the most popular criteria to confirm the diagnosis [5,8]. The EFNS/PNS criteria include clinical and electrodiagnostic criteria, as shown in Tables 3, 4 [9], respectively.

**Table 3:** Clinical diagnostic criteria. POEMS: polyneuropathy, organomegaly, endocrinopathy, monoclonal gammopathy, and skin changes; PNS: peripheral nervous system; CIDP: chronic inflammatory demyelinating polyneuropathy Table adapted from the Joint Task Force of the EFNS and the PNS [9].

Inclusion criteria:
(a) Typical CIDP:
Chronically progressive, stepwise, or recurrent symmetric proximal and distal weakness and sensory dysfunction of all extremities, developing over at least two months; cranial nerves may be affected; and
Absent or reduced tendon reflexes in all extremities
(b) Atypical CIDP (still considered CIDP but with different features): One of the following, but otherwise as in (a) (tendon reflexes may be normal in unaffected limbs):
Predominantly distal (distal acquired demyelinating symmetric),.or
Asymmetric [multifocal acquired demyelinating sensory and motor neuropathy, Lewis–Sumner syndrome], or
Focal (e.g., involvement of the brachial or lumbosacral plexus or of one or more peripheral nerves in one upper or lower limb),
Pure motor, or
Pure sensory (including chronic immune sensory polyradiculopathy affecting the central process of the primary sensory neuron)
Exclusion criteria:
*Borrelia burgdorferi* infection (Lyme disease), diphtheria, drug or toxin exposure probably causing the neuropathy
Hereditary demyelinating neuropathy
Prominent sphincter disturbance
Diagnosis of multifocal motor neuropathy
IgM monoclonal gammopathy with high titer antibodies to myelin-associated glycoprotein
Other causes for demyelinating neuropathy, including POEMS syndrome, osteosclerotic myeloma, and diabetic and non-diabetic lumbosacral radiocomplexes neuropathy. PNS lymphoma and amyloidosis may occasionally have demyelinating features

**Table 4:** Electrodiagnostic criteria. To apply these criteria, the median, ulnar (stimulated below the elbow), peroneal (stimulated below the fibular head), and tibial nerves on one side are tested. If the criteria are not fulfilled, the same nerves are tested at the other side, and/or the ulnar and median nerves are stimulated bilaterally at the axilla and the Erb’s point. Motor conduction block is not considered in the ulnar nerve across the elbow and at least 50% amplitude reduction between Erb’s point and the wrist is required for probable conduction block. Temperatures should be maintained to at least 33°C at the palm and 30°C at the external malleolus (good practice points). CMAP: compound muscle action potential; ULN: upper limit of normal values; LLN: lower limit of normal values; ^a^any nerve meeting any of the criteria (a–g) Table adapted from the Joint Task Force of the EFNS and the PNS [9].

(1) Definite: at least one of the following:
(a) Motor distal latency prolongation ≥50% above ULN in two nerves (excluding median neuropathy at the wrist from carpal tunnel syndrome), or
(b) Reduction of motor conduction velocity ≥30% below LLN in two nerves, or
(c) Prolongation of F-wave latency ≥30% above ULN in two nerves (≥50% if amplitude of distal negative peak CMAP <80% of LLN values), or
(d) Absence of F-waves in two nerves if these nerves have distal negative peak CMAP amplitudes ≥20% of LLN + ≥1 other demyelinating parameterᵃ in ≥1 other nerve, or
(e) Partial motor conduction block: ≥50% amplitude reduction of the proximal negative peak CMAP relative to distal, if distal negative peak CMAP ≥20% of LLN, in two nerves, or in one nerve + ≥1 other demyelinating parameterᵃ in ≥1 other nerve, or
(f) Abnormal temporal dispersion (>30% duration increase between the proximal and distal negative peak CMAP) in ≥2 nerves, or
(g) Distal CMAP duration (interval between onset of the first negative peak and return to baseline of the last negative peak) increase in ≥1 nerve (median ≥6.6 ms, ulnar ≥6.7 ms, peroneal ≥7.6 ms, tibial ≥8.8 ms) + ≥1 other demyelinating parameterᵃ in ≥1 other nerve
(2) Probable:
(a) ≥30% amplitude reduction of the proximal negative peak CMAP relative to distal, excluding the posterior tibial nerve, if distal negative peak CMAP ≥20% of LLN, in two nerves, or in one nerve + ≥1 other demyelinating parameterin ≥1 other nerve
(3) Possible:
(a) As in (1) but in only one nerve

In rare cases, CIDP can present as an extraintestinal manifestation of CD [10]. Several cases with CD have been reported with CIDP [10]. In some cases, patients developed CIDP between 1 and 30 years after the onset of CD, whereas other cases developed simultaneously, with one acute progressive episode that made the diagnosis difficult and undifferentiated from acute inflammatory demyelinating polyneuropathy [10]. A prospective study evaluated the electrodiagnostic and clinical findings for the presence of polyneuropathy in patients with CD and ulcerative colitis (UC) [11]. Both demyelinating and non-demyelinating neuropathies were observed [11]. Some CD patients presented with demyelinating neuropathy in the form of multifocal motor neuropathy and CIDP, whereas others presented with small to predominantly axonal sensory large fiber [11]. Similarly, UC patients presented with the demyelinating phenotype, whereas individuals with other phenotypes presented with small and large axonal neuropathy [11].

Infliximab is a monoclonal antibody used against TNF-α and has been approved for use in the treatment of several immune-mediated diseases. Infliximab is one of the biological agents that can be used for patients with active CD who failed to respond to immunomodulatory therapy [12-14]. Anti-TNF-α has been reported to be associated with the development of both CNS and PNS demyelination [15]. CIDP has been reported in several cases in association with infliximab use for different rheumatological diseases [16]. One case reported a patient with CD developing CIDP following the use of infliximab therapy [3]. However, the patient did not improve after discontinuation of infliximab and had a recurrence of his symptoms requiring further treatment. This case is similar to our patient as his symptoms developed in a stable CD setting, which makes it likely secondary to anti-TNF-α use.

The mechanism of developing CIDP with infliximab is not well understood. Several mechanisms are thought to be related to the development of demyelination in a setting of anti-TNF-α that include increased susceptibility to infection, induction of autoimmune process, and an imbalance between TNF-α and their receptors [4]. Our patient developed symptoms 12 months after the start of infliximab therapy in a stable CD setting. His conditions improved with immunotherapy. It is uncertain whether the development of his CIDP is related to infliximab therapy or related to the CD itself. However, the development of CIDP in a setting of stable CD makes it unlikely that it was related to the disease itself.

Different cases have reported that discontinuing infliximab may be ineffective in resolving neuropathy [3]. The improvement seen in our patient despite continuing treatment, the findings of nerve conduction studies, and the objective evidence confirming the development of CIDP in patients with CD support the conclusion that CIDP can be one of the extraintestinal manifestations of CD.

The management of CIDP needs a multidisciplinary approach requiring neurology, physical therapy, and occupational therapy. Pharmacological intervention is the mainstay to target inflammatory demyelination and the functional disability caused by the disease [7]. There are three approved first-line CIDP treatments, namely, corticosteroids, plasma exchange, and intravenous immunoglobulins [7]. Multiple second-line treatments can be used when the first-line treatment cannot be used due to side effects, inconvenience, or cost [7]. The treatment of CD associated with CIDP is similar to the treatment offered to patients with CIDP alone. Patients initially receive immunotherapy via oral or intravenous steroids, intravenous immunoglobulins, or plasma exchange after the exclusion of secondary causes and the diagnosis of peripheral neuropathy with CD [6].

Our patient was treated similar to other cases of CIDP, starting with steroids and then progressing to azathioprine. This yielded a significant improvement of his motor power despite being on infliximab for his CD. In patients with CD who present with progressive, stepwise, or recurrent symmetric proximal and distal weakness and sensory dysfunction of all extremities, developing over at least two months, a physical examination should be done and electrodiagnostic studies should be obtained to rule out CIDP.

## Conclusions

For patients with CD presenting with progressive weakness, an extensive workup is necessary. Moreover, it is important to rule out nutritional causes, medication side effects, and to consider CIDP as a possible etiology. CIDP should be ruled out with electrodiagnostic studies. Once CIDP is diagnosed, treatment should start, and patients should be followed up closely with neurological examinations. The treatment of CIDP in the setting of CD is similar to idiopathic cases.
